# Supplementary material for: Patterns of Intron Gain and Loss in Fungi
Source: PLoS Biol. 2004 Nov 30;2(12):e422. doi: 10.1371/journal.pbio.0020422 (PMC532390; doi:10.1371/journal.pbio.0020422)
Supplement: Table S1 — Also available at http://genes.mit.edu/NielsenEtAl/. (4.3 MB ZIP). [file pbio.0020422.st001.zip › NielsenEtAl/html/715.html]

AN1342.1.NCU01821.1.MG02525.1.FG10893.1


```
 CLUSTAL W (1.82) Multiple Sequence Alignments - Introns Inserted


Sequence 1: NCU01821.1	446 aa
Sequence 2: MG02525.1	385 aa
Sequence 3: FG10893.1	455 aa
Sequence 4: AN1342.1	391 aa
Alignment Length: 462 aa
Number Identitical Residues: 210 aa
Alignment Score (without introns) 10424


MG02525.1 	------------------------------------------------------------
NCU01821.1	--------MPRSSGPVDSPARLSLISRHLAPD-YPLNTPYTVERHPDNIDTSLLPKTQTR
FG10893.1 	MEGRKVPRVPELSTEGPSSAGFKTYSAAVSADRISLVSGHLSHQTSSYNAASPALSSATQ
AN1342.1  	------------------------------------------------------------
          	                                                            

MG02525.1 	----------MSSQPEHPTLLIPGPIEFDDAVLQ~-----SMSHYS2ESHVGPAFVNVFG
NCU01821.1	ALTTDTTPANMSTQPEHATLLIPGPIEFDDAVLN~-----SMSHYS2ESHVGPGFVAVFG
FG10893.1 	ARSLSTTTANMSSQPEHATLLIPGPIEFDDDVLK~-----SMGHYS2ESHVGPGFVNTFG
AN1342.1  	----------MSTQPAHPTLLIPGPIEFDDAVLH2PSTPTNLSITS~ESHVAPGFVKVFG
          	          **:** *.************ **: .::.:.:.  * ****.*.** .**

MG02525.1 	ETLTLLRKLFQTTDSGSQPFVISGSGTLGWDLVSANLVEAGEDVLVLGTGYFSDGFADCL
NCU01821.1	ETLSMLRKLFQTTDPSAQPFVLSGSGTLGWDLVAANLIEPGEDALVLSTGYFGDGFADCL
FG10893.1 	ETLSMTRKLFQSTDPSAQPYIISGSGTLGWDIVAANLVEAGEDALVLSTGYFGDGFADCL
AN1342.1  	ETLSMTRKLFQSTNPAAQPFVISGSGTLGWDFVASNLVEKGENALVLHSGYFADSFASCL
          	***:: *****:*:..:**:::*********:*::**:* **:.*** :***.*.**.**

MG02525.1 	RTYGANVTELKAPVGSRPQLPEIEKALSAKKYKVLTVTHVDTSTGVLSELKDLSAMVHKV
NCU01821.1	KVYGANVTQISAEPGSRPQLPEIEKALSEKKYKLLTVTHVDTSTGVLSELKNLSELVHRV
FG10893.1 	RAYGANVTKLDGEVGGRPQLPEIEKALSEKKYKILTVTHVDTSTGVLSELKNLAATVKRV
AN1342.1  	ETYGANATQLKAPIGERPSFEAIEQALKEKPYKIITITHVDTSTGVLSDIKTVAEIVRRV
          	..****.*::..  * **.:  **:**. * **::*:***********::* ::  *::*

MG02525.1 	SPETLIIADGVCSVASEEIAFDEWGLDGVITASQKAIGCPAGLSISMFSSRAIK-AFEAR
NCU01821.1	SPDTLVIVDGVCSVACEEIDFDAWKLDGVVTASQKAIGCPAGLSISMFSGRAIQ-AFQNR
FG10893.1 	SPETLVIVDGVCSVACEEIAFDEWGLDGVVTASQKAIGVPAGLSISFFSGRAVAAALENR
AN1342.1  	SPQTLVVVDGVCSVGCEEIAFDEWDLDVVLTASQKAIGCPPGLSIIMFSGRAIE-TFKSR
          	**:**::.******..*** ** * ** *:******** *.**** :**.**:  ::: *

MG02525.1 	KTPPASYFASMKNWLPI1MRNYEAKKPSYFATPSPQLIHALHTALTQIMSRPLAERFAKH
NCU01821.1	QTPPTSYFASMKNWLPI1MQNYEAKKPSYFATPSPQLVRALHTALGQIVAQGVKERFAAH
FG10893.1 	KTPIPAYFASMKNWTPI1MKNYEAKKPSYFATPSPQLVHALHTSLTQILSKPLSERFQGH
AN1342.1  	KTPPSSYYSSMANWLPI~MQNYENFKPSYFATPSTQLIHALHTTLSQITSRPMAERFAAH
          	:** .:*::** ** ** *:***  *********.**::****:* ** :: : ***  *

MG02525.1 	KEASSKIKKAIADLGLKQVASNPADQANGMTAVYLPESVKPTDILPKLFSKGVIFAGGIH
NCU01821.1	KAASDKIKAAVAALGLKQVAANPDDQAHGMTAIYLPENVKGAELLPKLASRGVVFAGGIH
FG10893.1 	IEVSNKVKKAITDLGLKIVATKPEDQAHAMTAIYLPEGVGAPDVLPKLAGKGVVFAGGIH
AN1342.1  	RRASDRVKAAVAELGLQQLASKVENQAHAMTAIWLPDGLAPPDVLPGLLKRGVIFAAGLH
          	  .*.::* *:: ***: :*::  :**:.***::**:.:  .::** *  :**:**.*:*

MG02525.1 	KEIAPKYIRFGHMGVSV0LNPERGDLDRAIKALESGLSECGYQKA-
NCU01821.1	KAIAAKYIRFGHMGVSV~LDPSRGHVEKAIKALEEGLSECGYQKA-
FG10893.1 	KAIASKYIRFGHMGVSA0LDPSRNHMEKAINALKESLFEAGYKA--
AN1342.1  	KEVATKYIRFGHMGVSI~MDPARDDIEKAIAALKEAIADAKRAKGL
          	* :*.***********  ::* *..:::** **:..: :.    .
```
